# Effects of Dietary Supplementation of Pomegranate Peel with Xylanase on Egg Quality and Antioxidant Parameters in Laying Hens

**DOI:** 10.3390/antiox12010208

**Published:** 2023-01-16

**Authors:** Styliani Lioliopoulou, Georgios A. Papadopoulos, Ilias Giannenas, Konstantina Vasilopoulou, Clare Squires, Paschalis Fortomaris, Fani Th. Mantzouridou

**Affiliations:** 1 Laboratory of Animal Husbandry, Faculty of Veterinary Medicine, Aristotle University of Thessaloniki, 541 24 Thessaloniki, Greece; 2 Laboratory of Animal Nutrition, Faculty of Veterinary Medicine, Aristotle University of Thessaloniki, 541 24 Thessaloniki, Greece; 3 Laboratory of Food Chemistry and Technology, School of Chemistry, Aristotle University of Thessaloniki, 541 24 Thessaloniki, Greece

**Keywords:** pomegranate, xylanase, diet, laying hens, egg quality

## Abstract

Pomegranate contains bioactive compounds in all its parts. In this study, two levels of pomegranate peel byproduct (PPB) with or without the inclusion of xylanase enzyme were used to supplement laying hens’ diet, in a 2 × 2 full factorial design. A total of 48 Isa brown laying hens were fed the following experimental diets for 8 weeks: T1 (2.5% PPB); T2 (2.5% PPB and xylanase); T3 (5% PPB); T4 (5% PPB and xylanase). Eggs collected were analyzed for egg quality parameters. Moreover, egg yolks were analyzed for Malondialdehyde content (MDA), fatty acid profile and total phenolic content. The T2 eggs showed enhanced yolk coloration and greater yolk total phenolic content. The T3 and T4 egg yolks showed lower MDA levels compared with T1, T2. Overall, results have shown that (a) xylanase inclusion affected egg yolk coloration and total phenolic content when combined with 2.5% PPB dietary supplementation; (b) dietary supplementation of 5% PPB resulted in eggs with reduced MDA levels.

## 1. Introduction

Pomegranate (*Punica granatum*) is a shrub or small tree cultivated widely in Mediterranean and Middle East regions. Pomegranate fruit is one of the world’s most ancient fruits, known for its antioxidant, anti-inflammatory, antimicrobial, antiatherogenic, anticancer and other beneficial properties [1,2,3,4,5,6]. It contains bioactive compounds in both edible and non-edible parts. The pomegranate processing industry produces a significant number of byproducts (leaves, peels, seeds) which are left as waste to decompose in the environment. These byproducts are rich in nutrients and could potentially be used as feed additives for livestock and hence support the circular economy. This could be an important cost-saving and environmentally-friendly strategy with beneficial effects for animal health and production, considering that such byproducts are rich in polyphenols (phenolic acids, tannins and flavonoids, particularly anthocyanins) [7].

In poultry production, previous studies have shown that pomegranate supplementation in the diet improved the health of the birds and the quality of their products. In particular, in studies with broilers, results showed positive effects on the immune system of birds, their performance and meat quality and altered intestinal microflora [8,9,10,11,12,13,14]. Moreover, there are studies where gas emissions were reduced as well as the incidence of ascites [9,11]. A study with Japanese quails showed that replacement of yellow corn with pomegranate peel powder at a level 7.5% can improve productive and physiological parameters and jejuna morphology [15]. In laying hens, Saki et al. (2014, 2019) [16,17] and Kostrogys et al. (2016) [18] investigated the effects of dietary supplementation of pomegranate seed pulp (up to 15%) and pomegranate seed oil, respectively. Their studies showed increased egg production and improvement of several egg quality parameters (egg mass, yolk color, yolk Fe content, yolk punicic acid and conjugated linoleic acid percentages). No negative effects on egg organoleptic characteristics were noticed in these studies.

In practice, exogenous enzymes are widely used as feed additives in livestock production. The notion is that enzymes increase the bioavailability of nutrients in the diet by breaking down specific bonds. They are also used to overcome antinutritional factors and substitute enzymes not secreted by the organs of the gastrointestinal tract of animals [19]. Monogastric animals lack the appropriate enzymes to break down the complex structures of plant cell walls, especially non-starch polysaccharides (NSP). The NSPs include celluloses, hemicelluloses, lignin, pectin, gums, and mucilages. Elevated levels of NSP in poultry diets increase intestinal content viscosity, resulting in slower digesta passage rate, increased fermentation by gut microflora and decreased nutrient digestion and absorption [20,21,22,23]. Xylanases are a group of depolymerizing enzymes that hydrolize xylan, a major component of hemicelluloses. The effects of xylanase supplementation in poultry is more apparent in diets rich in NSP, for example wheat-based diets. There is abundant evidence in the literature about the role of xylanase in laying hens’ diets. It increases productivity and nutrient absorption and hence reduces production costs [24,25,26,27]. Xylanase supplementation enhanced egg production, egg mass and feed efficiency in laying hens fed corn-SBM-dried distillers’ grains with solubles-based diets [26]. Another study [24] reached similar findings by using a commercial enzyme mixture containing xylanase. Moreover, it was found that the inclusion of xylanase in corn-soybean-meal-wheat-based diets of laying hens increased eggshell thickness, Haugh unit, albumen height and excreta lactic acid bacteria numbers [28]. In a recent study [29], xylanase supplementation improved eggshell thickness and Ca content in laying hens fed with an addition of modern hybrid rye to a wheat-corn diet. Bone strength was also improved by xylanase supplementation, irrespective of the dietary addition of the modern hybrid rye.

However, there is scarce evidence in the literature about combined dietary supplementation of pomegranate byproducts with xylanase. For example, in the study of Saki et al. (2014) [16], different levels of pomegranate seed pulp were used in laying hens’ diet, with the addition of a multi-enzyme mixture containing xylanase. However, all groups were fed the same amount of multi-enzyme mixture, so the results were inconclusive regarding the actual effects of the xylanase. Hence, the objective of our study was to investigate its actual role in health and productivity of laying hens fed a diet with different levels of pomegranate peel byproduct and xylanase. It was hypothesized that the inclusion of xylanase could possibly enhance the absorption of the bioactive compounds of pomegranate and therefore further improve egg quality.

## 2. Materials and Methods

### 2.1. Ethical Considerations

Experimental procedures were approved by the Ethical Committee branch of the Research Committee of Aristotle University of Thessaloniki, Greece (approval number 24828/2021). The animal phase of the experiment was designed considering all welfare requirements described by Good Farming Practice Guidelines [30].

### 2.2. Experimental Design

The study took place in a designated chamber of a commercial poultry house located in Galatista, a municipality of Chalkidiki, Greece. A total of 48 Isa Brown laying hens, about 45-weeks-old, were randomly assigned to four groups and fed on the following diets for 8 weeks: T1 (2.5% PPB); T2 (2.5%PPB and xylanase); T3 (5% PPB); T4 (5%PPB and xylanase). All diets were formulated according to Isa Brown commercial product guide [31]. Nutrient analysis of diets is presented in Table 1. Air-dried pomegranate peels were obtained from a local pomegranate juice industry and were ground to form a pomegranate peel powder. The nutritional analysis of pomegranate peel used in this study, is shown in Table 2. The enzyme product used was Ronozyme^®^ WX, a preparation of endo-1,4-β-xylanase produced by a genetically modified strain of *A. oryzae* (DSM 26372). It was added at the recommended dose of 100 g/t during the mixing procedure together with the premix of minerals and vitamins. Before the start of the experimental period, there was a 2-week adaptation period in which the birds were fed the basal diet without the designated supplements. The birds were individually placed in 40 cm × 40 cm cages, which ensure more area per hen (1600 cm^2^) than the minimum requirements of a recent EU directive (at least 750 cm^2^ of cage area per hen) [32]. The birds had access to feed and water ad libitum. Light duration was 14 h/day, based on the ISA Brown management guide [31]. Temperature was between 20–24 °C and relative humidity between 55–70%. Egg samples were collected at the end of the 8th week (24 eggs per treatment). The eggs were analyzed immediately for egg quality parameters and the yolks were stored in individual containers at −20 °C for further analysis.

### 2.3. Egg Quality Parameters

Eggs were analyzed for the following quality parameters: egg weight, yolk weight, albumen weight, eggshell weight, eggshell thickness, longitudinal and transverse axes, shape index, eggshell color, yolk color, Haugh units and specific gravity. Yolk color was scored with DSM YolkFanTM scale and measured instrumentally with Chroma meter CR-410 (Konica Minolta, Osaka, Japan) using the L*a*b* color values. The L* value represents lightness (0 = black, 100 = white), the a* value indicates redness (−100 = green, 100 = red) and the b* value indicates yellowness (−100 = blue, 100 = yellow).

### 2.4. Yolk Malondialdehyde (MDA) Content

The Malondialdehyde (MDA) content of egg yolk was measured by TBARS assay (Thiobarbituric Acid Reactive Substances). Briefly, 1 g of yolk was placed in a falcon tube with 8 mL of 5% Trichloroacetic acid (TCA) aqueous solution and 5 mL of 0.8% Butylated hydroxytoluene (BHT) solution in hexane and then homogenized using an Ultra-Turrax device (model T25-S5, IKA-Labortechnik, Janke & Kunkel, GMBH, Stuttgart, Germany) and a Vortex apparatus (REAX 1R model, Heidolph, Germany). The tubes were centrifuged at 3000 rpm for 3 min. Afterwards, 2.5 mL of the bottom layer was transferred in tubes with the addition of 1.5 mL of 0.8% Thiobarbituric Acid aqueous solution. The tubes were placed in a water bath at 70 °C for 30 min. Then, the tubes were cooled in tap water. Immediately after processing the samples, absorbance was measured in a spectrophotometer at 532 nm. The MDA content of egg yolk was expressed as ng MDA/g yolk.

### 2.5. Yolk Fatty Acid Profile

#### 2.5.1. Preparation of Fatty Acid Methyl Esters

The methodology used to process samples was according to O’Fallon et al. (2007) [33] and specifically: 1 g of sample was placed in a 15 mL glass tube with 5.3 mL of methanol (MeOH) and 0.7 mL of potassium hydroxide (KOH) (for the preparation of potassium hydroxide solution, 56.1056 g of caustic potassium was added in water to a final volume of 100 mL). Thereafter, samples were homogenized with an Ultra-Turrax device (model T25-S5, IKA-Labortechnik, Janke & Kunkel, GMBH, Stuttgart, Germany) for 20 s at 15,000 rpm. The next step was incubation in a water bath at 55 °C for 1.5 h with stirring every 20 min. The samples were removed from the water bath and cooled in cold water. The second phase of extraction involved the addition of 0.58 mL of a 24N solution of sulfuric acid (H_2_SO_4_) (66.5 mL of concentrated sulfuric acid in water to a final volume of 100 mL) and then the samples were stirred in a vortex apparatus (REAX 1R model, Heidolph, Germany). Subsequently, samples were incubated again in a water bath at 55 °C for 1.5 h with stirring every 20 min. After that, samples were removed from the water bath and cooled in cold water. Finally, 3 mL of hexane (C_6_H_14_) was added in the sample and it was stirred in a vortex apparatus. Finally, samples were centrifuged for 5 min and the top layer was transferred to 2 mL glass vials. Samples were placed in a freezer at −24 °C until Gas Chromatography (GC) analysis.

#### 2.5.2. GC Analysis

Fatty acid composition was determined by GC. The Gas Chromatographic system (TraceGC model K07332, ThermoFinnigan, ThermoQuest, Milan, Italy) used for methyl esters separation and quantification was equipped with a flame ionisation detector, a 5.01 version of Chrom-Card data system (Thermo Electron Corporation, Milan, Italy) and a fused silica capillary column, 30 m × 0.25 mm i. d., coated with cyanopropyl polysiloxane (phase type SP-2380) with a film thickness of 0.20 μm (Supelco, Bellefonte, PA, USA). The initial oven temperatutre was 37 °C, held for 4 min, subsequently increased at a rate of 4 °C/min to 250 °C for 5 min. The carrier gas was N2 at a flow rate of 1 mL/min. The other chromatographic conditions were: Inlet temperature: 250 °C; Detector temperature: 260 °C; Injection: 1 μL, with split 1/20. Fatty acid methylesters were identified by comparing their retention times and elution order with the Supelco ‘37 Component FAME Mix’ reference standard (Sigma-Aldrich). Fatty acids were quantified by peak area measurement. Results were expressed as percentage (%) of the total peak areas for all quantified acids.

### 2.6. Yolk Total Phenolic Content

The total phenolic content of each egg yolk was measured by the Folin–Ciocalteu assay, according to the protocol described by Shang et al. (2020) [34]. Egg yolks were dried in an oven at 80 °C for 2 h. Then, the oven temperature was increased at 130 °C and the samples were left in the oven for one night. The next morning, 2 g of dried yolk were weighed and placed in tubes with the addition of 8 mL MeOH(aq) 50%. The contents of the tubes were homogenized in a Vortex apparatus for 2 min and placed for centrifugation (3000× *g*, 20 min). Following centrifugation, 4 mL of the upper layer was transferred to a tube and 400 μL TCA 10% *w*/*v*, were added. The tubes were centrifuged in 3000× *g* for 20 min. The volume of the upper layer was measured and an aliquot of 125 μL was transferred into a glass tube with 125 μL of Folin–Ciocalteu reagent and 2.25 mL Na_2_CO_3_(aq) 7%. As Folin–Ciocalteu reagent is light sensitive, the procedures were performed under dim light conditions. A blank sample was also prepared with 125 μL MeOH(aq) 50%, 125 μL of Folin–Ciocalteu reagent and 2.25 mL Na_2_CO_3_(aq)7%. The tubes were covered and stored in darkness for 30 min. The absorption of the mixtures was assayed at 725 nm. The total phenolic content of egg yolk was measured as μg of Gallic Acid equivalent (GAE) per g of dried yolk using a Gallic Acid standard curve.

### 2.7. Statistical Analysis

Initially, two-way ANOVA was performed in SPSS 25.0 (IBM SPSS Statistics for Windows, Version 25.0. Armonk, NY, USA: IBM Corp.) to examine how the two independent variables (pomegranate level and presence or absence of xylanase), in combination, affect the measured parameters. The effect of dietary treatments on measured parameters was further analyzed with one-way ANOVA. The dietary treatment groups (T1, T2, T3, T4) were the fixed factors in the statistical model. Post hoc comparisons among treatments were investigated by Tukey’s test. The average values including the standard deviation of the mean were calculated for every examined parameter. The level of significance for all statistical evaluations was set at *p* < 0.05.

## 3. Results

### 3.1. Egg Quality Parameters

There were not significant differences between treatments regarding egg weight, yolk weight, albumen weight, eggshell weight and eggshell thickness (Table 3). The egg shape index was higher in the 2.5% group (*p* = 0.049 for PPB effect) and was significantly increased in the T2 group compared with T1 and T4 groups (*p* < 0.001 for interaction between PPB level and xylanase). An interaction effect was noted also for the longitudinal axis, which was higher in the T1 than the T2 group, while T3 and T4 had intermediate levels (*p* = 0.041). Eggshell color was lighter in the 5% PPB group (*p* < 0.001 for PPB effect) and in the group supplemented with enzyme (*p* = 0.004 for xylanase effect). Eggs from the 2.5% supplemented groups had higher Haugh units values in comparison with the 5% (*p* = 0.001 for PPB level). Egg specific gravity was higher in the 5% group (*p* = 0.001 for PPB effect) and was increased in the T3 group compared to T1 and T2 (*p* = 0.031 for interaction between PPB level and xylanase). The yolk color visual scoring showed significantly darker yolks in the T2 group compared with T1 and T4, because of the interaction of PPB level and xylanase supplementation (*p* < 0.001). The L* value was higher in the not supplemented with enzyme group (*p* = 0.005 for xylanase effect) and was the lowest in the T2 group (*p* = 0.043 for the interaction effect). The a* value showed the highest score in the T2 group, significantly different from the T4 group (interaction effect, *p* = 0.005). The b* value was significantly higher in egg yolks derived from groups supplemented with the higher 5% PPB inclusion level (*p* = 0.003).

### 3.2. Yolk Malondialdehyde (MDA) Content

The MDA levels in yolk were significantly lower in the 5% pomegranate groups compared to the 2.5% groups (*p* = 0.005 for PPB inclusion level effect), as shown in Table 4.

### 3.3. Yolk Fatty Acid Profile

No significant differences were detected in total saturated fatty acids (Σ SFA), total monounsaturated fatty acids (Σ MUFA), total polyunsaturated fatty acids (Σ PUFA), n6 and n3 of egg yolk, as shown in Table 5. The n6:n3 ratio increased in groups supplemented with 5% PPB (*p* = 0.027 for pomegranate level effect). There was also a reduction in arachidic acid percentage caused by the higher (5%) PPB inclusion level (*p* = 0.040 for pomegranate level effect). The xylanase inclusion reduced significantly (*p* = 0.014) the cis-11-Eicosenoic levels in yolk.

### 3.4. Yolk Total Phenolic Content

The egg yolk total phenolic content was expressed as μg of Gallic Acid equivalents per gram of dry yolk (μg GAE/g). There was an interaction between xylanase and PPB in 2.5% PPB inclusion level (*p* = 0.015) which increased egg yolk total phenolic content of T2 group. The results are presented in Table 6.

## 4. Discussion

Egg quality is a dominant factor determining consumers’ preference. As asserted in the Introduction, the main objective here was to assess quality parameters of eggs from hens fed diets with two levels of pomegranate peel byproduct with or without xylanase. A secondary objective was to address issues of the circular economy using such byproducts efficiently. The results showed that egg specific gravity and egg shape index were affected by PPB inclusion level; there was also a significant interaction between PPB and xylanase. These effects on eggshell characteristics are likely due to PPB content in Ca and P (Table 2) combined with Ca and P bioavailability subject to xylanase presence in the diet. Van der Klis et al. (1995) [35] found that xylanase supplementation affected positively the absorption of Ca in broilers fed a wheat-based diet. It is known that xylanases hydrolyze NSP in plant cell walls and may release encapsulated nutrients [36]. Moreover, many studies in broilers have shown that xylanase has a viscosity-reducing effect in birds’ digesta [37,38,39,40] which allows better nutrient digestibility, absorption, and bioavailability. In a recent study [29], xylanase supplementation in rye-wheat-corn diets improved eggshell quality and bone mineralization. Lei et al. [28] also suggested that xylanase improves the solubility and absorption of minerals, especially Ca, resulting in improved eggshell quality.

Egg yolk color is a key factor defining consumers’ preference. In European countries, the consumers prefer darker egg yolk colors [41]. In our study, the results from the visual scoring and Chroma meter values L* and a* showed that egg yolk color was enhanced; there was interaction between PPB and xylanase at the 2.5% PPB inclusion level. On the other hand, the b* value, representing the yellow hue, was only affected by the increased PPB inclusion level. Elsewhere, pomegranate seed oil affected positively egg yolk color [18], probably because of the presence of pomegranate pigments. The main pomegranate pigments responsible for its color are anthocyanins [42]; however, anthocyanins are water-soluble and eventually carotenoids, which are lipophilic molecules, have better deposition in egg yolk than anthocyanins. The possible underlying mechanism for our findings is that xylanase inclusion enhanced carotenoids’ absorption and deposition in egg yolk, which is in agreement with a previous study [43]. According to the latter, an indirect effect of xylanase on lipid metabolism could also affect positively the absorbance of carotenoids, as carotenoids are absorbed alongside with lipids by passive diffusion [44].

In brown-egg laying hens, protoporphyrin IX is the major pigment of eggshells, with traces of biliverdin and its zinc chelates also present [45]. In our study, the higher dose of PPB together with xylanase supplementation induced the production of lighter colored eggshells. It is known that protoporphyrin IX biosynthesis pathway begins from colorless molecules and ends with the auto-oxidation of the colorless molecule protoporphyrinogen to the brown colored pigment protoporphyrin [46]. It could be hypothesized that a higher inclusion level of PPB may have led to increased antioxidant absorption, resulting in a reduction of the auto-oxidation of protoporhyrinogen. Regarding the potential contibution of xylanase, the enzyme might have further enhanced the absorbance of antioxidants, as it affects beneficially nutrient bioavailability with the mechanism described previously. To our knowledge, this effect of pomegranate on eggshell color has not been previously reported. Regarding the xylanase effect on eggshell color, our findings agree with previous studies [47,48], which used enzyme mixtures containing xylanase and noticed lighter eggshell colorations. In another study [28], no significant change of eggshell color was observed in laying hens fed corn-soybean-meal-wheat-based diets supplemented with xylanase. This field warrants further investigation.

Haugh Units, a measure of egg albumen quality, were lower in the groups supplemented with 5% PPB. In previous studies [16,17,18] the researchers found no significant effects of dietary supplementation of pomegranate byproducts in laying hens on Haugh Units. The byproducts used in these studies were pomegranate seed oil, pomegranate seed pulp with multi-enzyme and a pomegranate peel byproduct up to 12%, respectively. The exact mechanism of our finding is not clear and further investigation is needed to propose a possible explanation.

Lipid oxidation is one of the main procedures that cause deterioration of animal product quality and shelf-life, causing adverse effects on organoleptic properties and nutritional value [49]. Malondialdehyde (MDA) has been pointed out as the main product to evaluate lipid peroxidation [50]. In this study, MDA levels were significant lower in the T3 group compared to T2, because of the higher PPB inclusion level, as shown by statistical analysis. The antioxidants contained in pomegranate peel are possibly involved, mainly flavonoids and hydrolizable tannins. This finding is of great importance, as this is the first in vivo study that measured MDA content of egg yolk following dietary supplementation of pomegranate in laying hens, to our knowledge. Several in vitro studies have showed that pomegranate could be used as a natural food preservative due to its antioxidant properties, as it reduces lipid peroxidation in meat products [50,51,52,53] and egg yolk [49].

No major changes were observed in egg yolk fatty acid profile among treatments, except for the n-6:n-3 ratio, which was significantly increased by the higher PPB inclusion level at 5%. The n-6:n-3 fatty acid ratio in the diet is an important factor for consumer health, as it is more important for the fat and cholesterol metabolism than absolute n6 or n3 [54,55,56]. In the study of Kostogrys et al. (2017) [18], increasing concentrations of punicic acid from pomegranate seed oils in laying hens’ diet increased the proportions of saturated fatty acids and decreased the proportions of monounsaturated and polyunsaturated fatty acids. Elsewhere, it was reported in women that consumption of pomegranate juice led to a significant increase in total MUFA content and a numerical increase of n-6:n-3 [57]. The latter authors postulated that polyphenols could prevent fatty acid oxidation, and as a result affect the fatty acid profile, by protecting especially unsaturated fatty acids which are prone to free radical damage [57]. It is plausible that a similar mechanism may have occurred in the present experiment, as the MDA levels were significantly lower in the 5% PPB group, in which the n-6:n-3 ratio was also found to be higher.

Total phenolic compounds are bioactive metabolites produced from plants. They can be transferred in egg yolk, as it was shown in a study which used dried tomato peel in laying hens’ diets [58]. In our study, a significant interaction between xylanase and PPB when it was supplemented at the 2.5% inclusion level tended to increase the total phenolic content of egg yolk. In other words, the xylanase effect on better absorbance and bioavailability of total phenolic compounds was more sufficient in PPB 2.5% inclusion level. The effectiveness of enzymatic hydrolysis is associated with the structural and chemical characteristics of the substrate [59]. The main substrate parameters that affect enzymatic hydrolysis effectiveness are cellulose crystallinity, degree of polymerization, lignin and hemicellulose content, and their distributions, particle size, and accessible surface area [60,61,62]. In diets containing the higher PPB inclusion level, the increased fiber content may affect xylanase effectiveness, and it can be assumed that in PPB above 2.5%, maybe the activity of xylanase reaches a plateau, due to the enzyme becoming saturated with substrate.

## 5. Conclusions

Dietary supplementation of pomegranate peel byproduct with or without xylanase affected egg quality characteristics. The results obtained indicate a favorable impact of supplementing laying hen diets at 5% PPB resulted in eggs on egg yolk MDA levels. Moreover, xylanase inclusion combined with 2.5% PPB resulted in improved total phenolic content. Further research is warranted to confirm the current findings and clarify the potential mechanisms involved.

## Figures and Tables

**Table 1:** Main ingredients of diets. Diets T2 and T4 contained Ronozyme^®^ WX 100 g/t.

Ingredients (%)	PPB 2.5%	PPB 5%
Corn	44.4353	45.0043
Soybean 47%	21.3382	21.3486
Wheat soft	6.0000	6.0000
Vetch byproduct	4.4354	4.0000
Ca 500/1200	5.6433	5.5988
Wheat bran	2.5000	6.0000
Barley	4.0000	4.0000
Ca 1200/2000	3.0000	3.0000
Molasses	3.0000	3.0000
Soybean oil	1.7604	1.6440
Nutriphos^®^ 22%	0.3027	0.2858
Sodium chloride	0.2028	0.2037
Vitamin premix	0.2000	0.2000
Mineral premix	0.2000	0.2000
D,L-Methionine	0.1828	0.1888
AdisodiumTM	0.1500	0.1500
Fish meal 65%	0.1000	0.1000
Calcium formate	0.0300	0.0300
Yeast	0.0100	0.0100
Pomegranate peel	2.5000	5.0000
Ronozyme^®^ HiPhos	0.0090	0.0090

**Table 2:** Chemical composition of pomegranate peel byproduct used in this study.

Parameter	Result
Moisture (g/100 g)	52.8
Ash (g/100 g)	0.10
Proteins (g/100 g)	4.9
Fat (g/100 g)	1.2
Crude Fibers (g/100 g)	12.1
Carbohydrates (g/100 g)	27.3
Energy (Kcal/100 g)	140
Ca (mg/kg)	1377
P (mg/kg)	1056

**Table 3:** Effects of two levels of pomegranate peel byproduct with or without xylanase on egg quality parameters. T1: 2.5% PPB; T2: 2.5% and xylanase; T3: 5% PPB; T4:5% PPB and xylanase. Values are means ± SD (*n* = 24 per treatment).

Treatments					Pomegranate Level		Xylanase Supplementation		*p*-Values		
Parameter	T1	T2	T3	T4	2.5%	5%	No Enzyme	Enzyme	Pomegranate	Xylanase	Pomegranate Level × Xylanase
Egg weight (g)	61.9 ± 6.76	60.3 ± 5.56	61.4 ± 5.19	60.0 ± 7.96	60.9 ± 6.00	61.1 ± 5.87	61.6 ± 5.75	60.2 ± 6.08	0.755	0.252	0.973
Yolk weight (g)	15.7 ± 1.49	16.1 ± 1.43	16.2 ± 1.76	17.3 ± 4.93	16.0 ± 1.45	16.4 ± 2.82	16.0 ± 1.67	16.4 ± 2.60	0.087	0.108	0.438
Albumen weight (g)	39.9 ± 4.78	38.5 ± 4.48	38.8 ± 3.71	36.9 ± 4.42	39.0 ± 4.59	38.3 ± 3.93	39.2 ± 4.13	38.1 ± 4.48	0.163	0.083	0.791
Eggshell weight (g)	6.1 ± 1.13	5.7 ± 0.85	6.1 ± 0.73	5.8 ± 0.94	5.8 ± 0.96	6.1 ± 0.79	6.1 ± 0.89	5.7 ± 0.86	0.733	0.078	0.879
Eggshell thickness (in)	0.018 ± 0.0022	0.018 ± 0.0019	0.019 ± 0.0016	0.018 ± 0.0022	0.018 ± 0.0020	0.019 ± 0.0018	0.019 ± 0.0019	0.018 ± 0.0019	0.423	0.055	0.685
Longitudinal axis (mm)	58.5 ± 2.23 ^a^	57.1 ± 1.87 ^b^	57.8 ± 1.61 ^ab^	58.3 ± 3.03 ^ab^	57.6 ± 2.09	57.9 ± 1.98	58.1 ± 1.86	57.4 ± 2.19	0.617	0.290	0.041
Transverse axis (mm)	43.8 ± 1.62	43.8 ± 1.44	43.8 ± 1.26	43.1 ± 1.82	43.8 ± 1.49	43.7 ± 1.42	43.8 ± 1.39	43.7 ± 1.42	0.261	0.285	0.252
Shape index	75.1 ± 2.25 ^bc^	76.7 ± 1.99 ^a^	75.9 ± 1.57 ^ab^	74.1 ± 2.91 ^c^	76.1 ± 2.21 ^a^	75.5 ± 2.06 ^b^	75.6 ± 1.87	76.1 ± 2.45	0.049	0.856	<0.001
Eggshell color	25.5 ± 6.72	27.6 ± 7.05	30.3 ± 7.70	37.2 ± 2.21	26.9 ± 6.95 ^b^	31.8 ± 7.42 ^a^	28.6 ± 7.66 ^b^	29.7 ± 7.46 ^a^	<0.001	0.004	0.114
Haugh units	74.2 ± 6.46	78.8 ± 9.67	65.2 ± 11.48	65.6 ± 10.69	76.4 ± 8.28 ^a^	65.4 ± 10.79 ^b^	69.9 ± 10.06	71.9 ± 12.03	0.001	0.405	0.490
Specific gravity	1.08 ± 0.007 ^b^	1.08 ± 0.010 ^b^	1.09 ± 0.014 ^a^	1.08 ± 0.014 ^ab^	1.08 ± 0.009 ^b^	1.09 ± 0.014 ^a^	1.09 ± 0.014	1.08 ± 0.011	0.001	0.186	0.031
Yolk color fan score	5.8 ± 0.77 ^b^	6.6 ± 0.82 ^a^	6.4 ± 0.65 ^ab^	5.8 ± 0.87 ^b^	6.3 ± 0.88	6.3 ± 0.74	6.2 ± 0.75	6.4 ± 0.88	0.645	0.570	<0.001
L*	82.9 ± 2.18 ^a^	80.0 ± 2.02 ^b^	82.8 ± 1.27 ^a^	82.3 ± 1.73 ^a^	81.5 ± 2.52	82.5 ± 1.51	82.9 ± 1.74 ^a^	81.2 ± 2.16 ^b^	0.070	0.005	0.043
a*	3.5 ± 1.38 ^ab^	4.4 ± 1.18 ^a^	3.9 ± 0.84 ^ab^	2.6 ± 1.28 ^b^	4.0 ± 1.33	3.2 ± 1.26	3.7 ± 1.13	3.5 ± 1.51	0.067	0.560	0.005
b*	53.2 ± 6.90	52.1 ± 5.48	59.3 ± 4.24	58.3 ± 7.46	52.7 ± 6.09 ^b^	58.7 ± 6.02 ^a^	56.2 ± 6.38	55.4 ± 7.16	0.003	0.601	0.987

^a,b,c^ Mean values in the same row and under the same grouping category (Treatments; Pomegranate level; xylanase supplementation) with a different superscript differ significantly (*p* ≤ 0.05).

**Table 4:** Effects of two levels of pomegranate peel byproduct with or without xylanase on egg yolk Malondialdehyde (MDA) content egg. T1: 2.5% PPB; T2: 2.5% and xylanase; T3: 5% PPB; T4:5% PPB and xylanase.

Treatments					Pomegranate Level		Xylanase Supplementation		*p*-Values		
Parameter	T1	T2	T3	T4	2.5%	5%	No Enzyme	Enzyme	Pomegranate Effect	Xylanase Effect	Pomegranate Level × Xylanase Effect
MDA (ng/g yolk)	47.1 ± 29.31	63.6 ± 48.35	27.1 ± 7.02	31.0 ± 11.09	55.4 ± 40.01 ^a^	29.1 ± 9.37 ^b^	38.0 ± 23.99	48.1 ± 38.76	0.005	0.258	0.487

^a,b^ Mean values in the same row and under the same grouping category (Treatments; Pomegranate level; xylanase supplementation) with a different superscript differ significantly (*p* ≤ 0.05).

**Table 5:** Effects of two levels of pomegranate peel byproduct with or without xylanase on egg yolk fatty acid profile. T1: 2.5% PPB; T2: 2.5% and xylanase; T3: 5% PPB; T4:5% PPB and xylanase. Values are means ± SD (*n* = 12 per treatment).

Treatments					Pomegranate Level		Xylanase Supplementation		*p*-Values		
Parameter	T1	T2	T3	T4	2.5%	5%	No Enzyme	Enzyme	Pomegranate	Xylanase	Pomegranate Level × Xylanase
Palmitic (C16:0)	31.48 ± 1.427	31.60 ± 0.798	30.92 ± 0.807	31.40 ± 0.758	31.54 ± 1.104	31.16 ± 0.787	31.20 ± 1.143	31.50 ± 0.750	0.357	0.462	0.668
Palmitoleic (C16:1)	4.35 ± 0.463	4.40 ± 0.748	4.55 ± 0.715	4.96 ± 1.424	4.38 ± 0.619	4.76 ± 1.103	4.47 ± 0.606	4.68 ± 1.131	0.316	0.550	0.629
Oleic (C18:1n9c)	39.97 ± 2.036	38.32 ± 2.266	39.84 ± 0.559	39.44 ± 2.155	39.14 ± 2.241	39.61 ± 1.640	39.92 ± 1.542	38.88 ± 2.204	0.529	0.201	0.427
a-Linolenic (C18:3n3)	0.49 ± 0.133	0.49 ± 0.116	0.47 ± 0.085	0.44 ± 0.054	0.49 ± 0.120	0.46 ± 0.073	0.48 ± 0.107	0.47 ± 0.096	0.421	0.703	0.654
Arachidonic (C20:4n6)	1.68 ± 0.210	1.64 ± 0.152	1.54 ± 0.061	1.56 ± 0.179	1.66 ± 0.177	1.55 ± 0.133	1.61 ± 0.165	1.60 ± 0.165	0.104	0.874	0.618
cis-4,7,10,13,16,19-Docosahexaenoic (C22:6n3)	0.50 ± 0.076	0.54 ± 0.089	0.51 ± 0.036	0.47 ± 0.075	0.52 ± 0.082	0.49 ± 0.059	0.50 ± 0.053	0.50 ± 0.087	0.227	0.952	0.197
Linoleic (C18:2n6c)	14.53 ± 1.437	14.53 ± 2.344	13.56 ± 1.559	13.38 ± 1.396	14.53 ± 1.900	13.47 ± 1.425	14.01 ± 1.525	13.95 ± 1.948	0.128	0.897	0.890
Stearic (C18:0)	7.08 ± 1.192	7.16 ± 0.598	6.66 ± 0.582	6.29 ± 0.841	7.12 ± 0.907	6.47 ± 0.722	6.87 ± 0.927	6.72 ± 0.835	0.054	0.650	0.480
Myristic (C14:0)	0.54 ± 0.161	0.50 ± 0.081	0.49 ± 0.047	0.60 ± 0.152	0.52 ± 0.124	0.55 ± 0.126	0.52 ± 0.120	0.55 ± 0.129	0.528	0.428	0.137
Myristoleic (C14:1)	0.12 ± 0.027	0.11 ± 0.030	0.11 ± 0.018	0.14 ± 0.058	0.115 ± 0.028	0.13 ± 0.045	0.12 ± 0.022	0.13 ± 0.047	0.419	0.479	0.212
Pentadecanoic (C15:0)	0.07 ± 0.009	0.07 ± 0.014	0.06 ± 0.010	0.07 ± 0.011	0.07 ± 0.012	0.07 ± 0.010	0.07 ± 0.011	0.07 ± 0.012	0.352	0.618	0.163
Heptadecanoic (C17:0)	0.13 ± 0.032	0.15 ± 0.035	0.14 ± 0.026	0.14 ± 0.023	0.14 ± 0.034	0.14 ± 0.023	0.13 ± 0.028	0.14 ± 0.030	0.719	0.381	0.430
cis-10-Heptadecenoic (C17:1)	0.13 ± 0.022	0.12 ± 0.019	0.13 ± 0.026	0.12 ± 0.046	0.12 ± 0.020	0.13 ± 0.036	0.13 ± 0.023	0.12 ± 0.034	0.622	0.354	0.950
Elaidic (C18:1n9t)	0.09 ± 0.032	0.06 ± 0.015	0.06 ± 0.038	0.07 ± 0.038	0.08 ± 0.027	0.07 ± 0.036	0.08 ± 0.035	0.07 ± 0.028	0.554	0.558	0.243
Linolelaidic (C18:2n6t)	0.007 ± 0.0034	0.003 ± 0.0017	0.006 ± 0.0035	0.005 ± 0.0049	0.005 ± 0.0033	0.006 ± 0.0040	0.006 ± 0.0034	0.004 ± 0.0036	0.859	0.143	0.299
γ-Linolenic (C18:3n6)	0.11 ± 0.011	0.12 ± 0.029	0.10 ± 0.031	0.10 ± 0.015	0.12 ± 0.021	0.10 ± 0.024	0.11 ± 0.024	0.11 ± 0.024	0.136	0.997	0.757
Arachidic (C20:0)	0.02 ± 0.007	0.01 ± 0.004	0.01 ± 0.004	0.01 ± 0.008	0.02 ± 0.007 ^a^	0.01 ± 0.006 ^b^	0.02 ± 0.007	0.01 ± 0.006	0.040	0.093	0.114
cis-11-Eicosenoic (C20:1n9c)	0.12 ± 0.030	0.07 ± 0.034	0.08 ± 0.036	0.06 ± 0.035	0.09 ± 0.041	0.07 ± 0.035	0.10 ± 0.039 ^a^	0.07 ± 0.033 ^b^	0.079	0.014	0.150
Henicosanoic (C21:0)	0.006 ± 0.0013	0.006 ± 0.0023	0.005 ± 0.0014	0.006 ± 0.0019	0.006 ± 0.0018	0.005 ± 0.0019	0.005 ± 0.0013	0.006 ± 0.0021	0.347	0.254	0.895
cis-11,14-Eicosadienoic (C20:2)	0.09 ± 0.017	0.09 ± 0.014	0.09 ± 0.012	0.07 ± 0.022	0.09 ± 0.016	0.08 ± 0.018	0.09 ± 0.014	0.08 ± 0.021	0.173	0.655	0.103
cis-11,14,17-Eicosatrienoic (C20:3n3)	0.10 ± 0.046	0.14 ± 0.032	0.11 ± 0.009	0.12 ± 0.008	0.12 ± 0.043	0.11 ± 0.010	0.10 ± 0.033	0.13 ± 0.027	0.514	0.057	0.263
Nervonic (C24:1n9)	0.08 ± 0.043	0.10 ± 0.016	0.10 ± 0.012	0.08 ± 0.010	0.09 ± 0.033	0.09 ± 0.013	0.09 ± 0.033	0.09 ± 0.016	0.939	0.756	0.084
Σ SFA	38.71 ± 1.524	39.13 ± 1.005	38.59 ± 0.515	38.95 ± 0.984	38.92 ± 1.259	38.78 ± 0.793	38.66 ± 1.129	39.04 ± 0.960	0.723	0.367	0.942
Σ MUFA	44.83 ± 1.736	43.18 ± 2.558	44.71 ± 1.573	44.52 ± 0.612	44.00 ± 2.270	44.62 ± 1.185	44.77 ± 1.593	43.79 ± 1.978	0.387	0.193	0.303
Σ PUFA	16.45 ± 2.751	17.70 ± 2.801	16.91 ± 1.136	16.16 ± 1.481	17.07 ± 2.744	16.50 ± 1.337	16.66 ± 2.092	16.93 ± 2.296	0.529	0.775	0.254
n6	15.31 ± 2.530	16.38 ± 2.544	15.70 ± 1.083	15.06 ± 1.317	15.85 ± 2.500	15.35 ± 1.211	15.49 ± 1.931	15.72 ± 2.063	0.555	0.785	0.284
n3	1.06 ± 0.301	1.21 ± 0.266	1.12 ± 0.057	1.02 ± 0.154	1.14 ± 0.285	1.07 ± 0.125	1.08 ± 0.218	1.12 ± 0.232	0.448	0.717	0.154
n6:n3	13.48 ± 1.182	13.69 ± 0.333	14.0 ± 0.555	14.86 ± 1.079	13.57 ± 0.869 ^b^	14.43 ± 0.938 ^a^	13.76 ± 0.899	14.37 ± 1.021	0.027	0.147	0.367

^a,b^ Mean values in the same row and under the same grouping category (Treatments; Pomegranate level; xylanase supplementation) with a different superscript differ significantly (*p* ≤ 0.05).

**Table 6:** Effects of two levels of pomegranate peel byproduct with or without xylanase on egg yolk total phenolic content. T1: 2.5% PPB; T2: 2.5% and xylanase; T3: 5% PPB; T4:5% PPB and xylanase. Values are means ± SD (*n* = 12 per treatment).

Treatments					Pomegranate Level		Xylanase Supplementation		*p*-Values		
Parameter	T1	T2	T3	T4	2.5%	5%	No Enzyme	Enzyme	Pomegranate Effect	Xylanase Effect	Pomegranate Level × Xylanase Effect
μg GAE/g dry yolk	135.1 ± 24.44 ^b^	164.4 ± 41.92 ^a^	148.2 ± 25.90 ^ab^	133.2 ± 31.73 ^b^	149.8 ± 36.79	140.7 ± 29.39	141.7 ± 25.55	148.8 ± 39.75	0.308	0.421	0.015

^a,b^ Mean values in the same row and under the same grouping category (Treatments; Pomegranate level; xylanase supplementation) with a different superscript differ significantly (*p* ≤ 0.05).

## Data Availability

The data presented in this study are available within the article.
